# RKIP does not contribute to MAP kinase pathway silencing in the Merkel Cell Carcinoma cell line UISO

**DOI:** 10.1186/1477-3163-6-16

**Published:** 2007-10-24

**Authors:** Roland Houben, Sonja Ortmann, Juergen C Becker

**Affiliations:** 1 Klinik und Poliklinik für Dermatologie, Venerologie und Allergologie, Julius-Maximilians-Universität, Josef-Schneider-Str. 2, D-97080 Würzburg, Germany

## Abstract

**Background:**

The Raf kinase inhibitor protein (RKIP) has been shown to block MAP kinase pathway as well as NFκB signalling. By means of immunohistochemistry, we previously demonstrated that the MAP kinase pathway is virtually inactive in Merkel cell carcinoma (MCC). Similarly to MCC *in situ *high RKIP expression accompanies absence of ERK phosphorylation in the MCC cell line UISO suggesting that RKIP might be causative for MAP kinase pathway silencing.

**Methods:**

Applying an siRNA approach RKIP expression was knocked down in UISO cells and a possible influence on MAP kinase pathway activity was assessed by Western blot analysis using phospho-specific antibodies. Moreover, a possible effect of RKIP knock down in UISO cells on proliferation as well as chemosensitivity to cisplatin were examined applying the MTS assay.

**Results:**

Surprisingly the absence of phosphorylation of the MAP kinases ERK1 and ERK 2 even following growth factor stimulation was not affected by the RKIP knock down indicating that RKIP is not essential for blocking the MAP kinase pathway in the MCC cell line UISO. Moreover, proliferation as well as chemosensitivity towards cisplatin were not altered upon knock down of RKIP.

## Introduction

Merkel cell carcinoma (MCC) is a highly aggressive skin cancer of neuroendocrine origin [1]. The tumors commonly affect elderly patients and are frequently located in body areas that are chronically sun exposed [2]. The genetic and molecular mechanisms involved in the development and progression of MCC are largely unknown.

A signal transduction pathway that is activated in many tumor entities is the MAP kinase pathway, a major regulator of cell growth, differentiation and survival. It comprises the three consecutive kinases Raf, MEK (MAP kinase kinase) and ERK (p42/p44 MAP kinases) which are controlled by the small G-Protein Ras [3]. Recently, we demonstrated by immunohistological analysis of 49 MCC tumors for expression and phosphorylation of ERK that the MAPK pathway is virtually inactive in MCC [4]. This inactivity is preserved in the MCC cell line UISO and activation of the MAPK pathway by an inducible Raf kinase in UISO cells induces apoptosis [5], possibly explaining why the pathway is generally shut off in MCC. A negative regulator of the MAP kinase pathway which can bind either to Raf or to MEK and thereby interfere with the activation of MEK is the Raf Kinase Inhibitor Protein (RKIP) [6]. In MCC *in situ *the absence of MAP kinase activation is accompanied by high level expression of RKIP [4].

## Materials and methods

### Cell culture

The MCC cell line UISO, which has been established from a primary Merkel cell carcinoma of a 46 year old woman [7], was grown in RPMI 1640 supplemented with 10% FBS, 100 U/ml penicillin and 0.1 mg/ml streptomycin.

### siRNA transfection

An Alexa Fluor 488 labelled scrambled siRNA as negative control (AAT TCT CCG AAC GTG TCA CGT) as well as an siRNA targeting RKIP (AAG GTG GCG TCC TTC CGT AAA) [8] were purchased from Qiagen (Hilden, Germany). 1.4 × 10^5 ^UISO cells were seeded in 24 well plates the day prior to transfection. Two different transfection reagents were used. The siRNA were transfected at 80 nM (Lipofectamine 2000; Invitrogen, Karlsruhe, Germany) or 10 nM (HiPerFect; Quiagen, Hilden, Germany) concentration according to the manufacturer's protocols.

### Proliferation and chemosensitity measured by the MTS assay

24 hours following siRNA transfection the cells were harvested with Trypsin/EDTA and were seeded with an equivalent of 2000 cells/well corresponding to the initial cell number in 96 well plates. For measurement of chemosensitivity cisplatin was added at this time point. To extend the period of high siRNA levels in the cells a second transfection using HiPerFect (Quiagen) was performed on day 3 following the first transfection. Another 3 days later proliferation and cell viability were assessed by the MTS assay (CellTiter 96^® ^AQueous One Solution Cell Proliferation assay, Promega Corporation, Madison, WI, USA). To this end, 10 μl of CellTiter 96^® ^AQueous One Solution Reagent containing a tetrazolium compound (MTS) were added to each well and the cells were incubated for approximately 90 min at 37°C. Metabolically active, viable cells convert MTS into a colored formazan product that was measured in a spectrophotometric microplate reader (Perkin-Elmer Inc., MA, USA) at 493 nm.

### Western blot analysis

For protein analysis cells were lysed using Laemmli buffer. Cell lysates were resolved by SDS-Polyacrylamid gel electrophoresis and transferred to nitrocellulose membranes. Following blocking for 1 h with phosphate-buffered saline containing 0.05% Tween 20 and 5% powdered skim milk, blots were incubated for 2 h or overnight with primary antibody, washed three times with phosphate-buffered saline supplemented with 0.05% Tween 20, and then incubated with the peroxidase coupled secondary antibody. The bands were detected using a chemo luminescence detection kit (Roche Diagnostics, Mannheim, Germany). The antibodies used were the monoclonal antibodies α-Phospho-p44/42 MAP kinase (Thr202/Tyr204) (clone E10; Cell Signaling, Beverly, USA), α-β-tubulin (Sigma, Ottobrunn, Germany) and the polyclonal antibody α-RKIP (Upstate, Charlottesville, USA).

## Results and discussion

**Figure 1:**
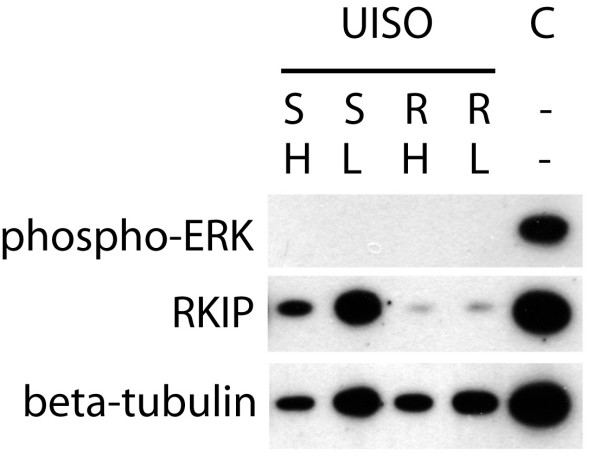
**RKIP *knock down *in UISO cells does not result in ERK phosphorylation**. UISO cells were transfected either with a scrambled siRNA (S) as negative control or with an siRNA targeting RKIP (R). Two different transfection reagents (i.e. Lipofectamine 2000 (L) and HiPerfect (H)) were used. 72 h following transfection total cell lysates were analysed by western blot using a phospho-ERK specific antibody. Untransfected MCC13 cells served as positive control (C) for ERK phosphorylation and probing for β-tubulin was used to visualize protein loading.

**Figure 2:**
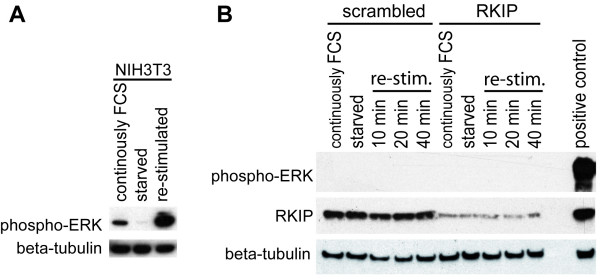
**RKIP *knock down *in UISO cells does not confer serum responsiveness of the MAPK pathway**. **(A) **NIH3T3 cells were cultured either in the presence of 10% FCS, or for 24 hours in the absence of FCS (starved), or were re-stimulated with 10% FCS for 20 min following starvation. Total cell lysates were subjected to Western Blot analysis and probed with the indicated antibodies. **(B) **UISO cells were transfected either with a scrambled siRNA (S) as negative control or with a siRNA targeting RKIP. Cells were harvested 72 hours later or when indicated FCS was withdrawn after 48 hours and following 24 hours of starvation the cells were restimulated with 10% FCS for the indicated time. Total cell lysates were subjected to Western Blot analysis and probed with the indicated antibodies. As positive control for phospho-ERK a lysate from the melanoma cell line SKmel-28 was used.

The MCC cell line UISO displays the same MAP kinase pathway phenotype as it is observed for Merkel cell carcinoma *in situ*, in particular the complete lack of ERK 1/2 phosphorylation associated with high expression of RKIP [4]. Remarkably, ERK remains unphosphorylated even after growth factor stimulation. We therefore speculated that the MAP kinase pathway may be kept silent through the action of RKIP, which can interfere with either the activation of C-Raf or with the activation of MEK by B-Raf or C-Raf [6,8]. Consequently, we used an RKIP knock down approach to test this notion. Using two different reagents for transfection of UISO cells with an siRNA targeting RKIP, in both cases greatly reduced RKIP protein levels compared to cells transfected with an scrambled siRNA not targeting any mRNA encoded by the human genome. Strikingly, this knock down however, did not induce any detectable phosphorylation of ERK (Figure 1). Even upon prolonged exposure of the films absolutely no phospho-ERK signal was detectable. When cells are starved from growth factors e.g. by FCS deprivation and are then re-stimulated, a very strong transient activation of ERK is observed (Figure 2A). Even under such conditions the RKIP knock down did not result in a detectable ERK phosphorylation. Therefore, the level of RKIP expression is not critical for the inactivity of the MAP kinase pathway in MCC cells.

The importance of the Ras/Raf/MEK/ERK signalling pathway for carcinogenesis is reflected by the facts that *Ras *genes are the most frequently mutated oncogenes detected in human cancer [9,10] and that B-Raf is activated in several malignancies with the highest frequency found in melanoma [11]. In MCC, however, we and others did not detect any B-Raf or Ras mutations [4,12,13].

In contrast, in MCC *in situ *as well as in the MCC cell line UISO MAP kinase pathway activity as measured by ERK phosphorylation is not detectable at all (Houben et al, 2006). Since activation of the MAP kinase pathway in UISO cells induces apoptosis [5], demonstrating that this is a negative selection factor for these MCC cells, it is plausible that inactivity has to be tightly regulated. In the UISO cells as well as in the MCC tissues, the lack of MAPK signalling is associated with high expression of the Raf kinase inhibitor protein (RKIP) [4]. RKIP is a molecule which was shown to block both MAP kinase pathway as well as NFκB signalling [14]. Therefore, it was tempting to speculate whether RKIP might be causative for MAPK pathway inactivity in MCC cells. Our observation, however, that even the almost complete reduction in RKIP protein expression by means of RNAi does not lead to ERK phosphorylation argues against this model. Still, RKIP is thought to interfere with the above mentioned signal transduction pathways by directly binding to particular pathway proteins. In case of the MAPK pathway, RKIP was shown to bind B-Raf and c-Raf as well as MEK and thereby inhibiting the Raf/MEK interaction [6]; in case of the NFκB pathway, NFκB-inducing kinase and transforming growth factor beta-activated kinase-1 are the targets. Therefore, the relative stochiometry of RKIP, not bound to other targets, and Raf/MEK should be critical for the ability of RKIP to completely block Raf mediated MEK activation. Moreover, RKIP itself is regulated by phosphorylation through Protein kinase C and only unphosphorylated RKIP binds to Raf [15]. Consequently, the absolute expression level of RKIP is only one parameter for RKIP activity. However, we recently demonstrated that MAPK pathway signalling is induced both in UISO-BXB-ER cells by activating a hormone regulatable Raf kinase and in UISO cells by the Raf activating agent ZM336372 [5]. Together with the observation that ERK phosphorylation in UISO cells cannot be induced by growth factor stimulation [4], these findings suggest that silencing of the MAPK pathway in MCC cells occurs upstream or at the level of Raf, hence it further argues against RKIP being responsible for the observed MAPK pathway inactivity in MCC.

**Figure 3:**
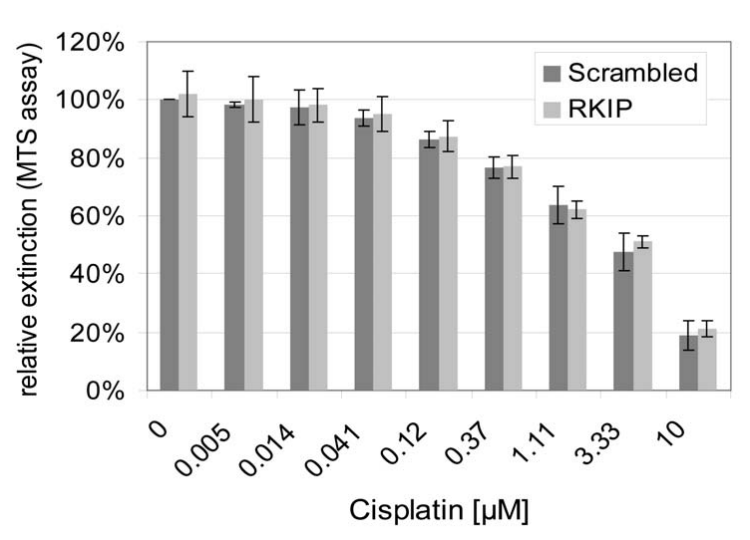
**RKIP *knock down *in UISO cells does not alter proliferation properties or chemosensitivity**. A first siRNA transfection was performed in 24 well plates with the indicated siRNA. 24 following transfection cells were split to 96 well plates and cisplatin was added as indicated. A second siRNA transfection was performed on day 3 following the first transfection. On day 6 proliferation and apoptosis were assessed using the MTS assay. Relative extinctions were calculated with the scrambled/no cisplatin sample set to 100%. Given are the mean values (± SD) of three independent experiments.

RKIP has been reported to be involved in the regulation of proliferation and apoptosis [16-18]. However, in these cases the effects of RKIP up- or down-regulation on growth or survival were always accompanied by changes in MAPK pathway signalling. Therefore we asked whether in UISO cells the level of RKIP protein expression might influence proliferation or apoptosis in a MAPK pathway independent fashion. To this end, we assessed proliferation of UISO cells following siRNA knock down of RKIP by means of the MTS assay. This analysis revealed essentially no differences between the cells in which RKIP was silenced or not (Figure 3, the first two columns). In order to test the impact of RKIP silencing on apoptosis in response to genotoxic stress cells were incubated with varying amount of cisplatin. Titration of this cytotoxic drug to the cells resulted in an increase of dead cells in the culture (data not shown) and in reduced signals in the MTS assay. However, again no differences were detectable whether RKIP expression was knocked down or not. Since the knock down of RKIP in UISO cells is not associated with elevation of ERK phosphorylation these findings support the view that the growth inhibiting and the apoptosis suppressing functions observed in other model systems can be attributed to the ability of RKIP to regulate MAPK pathway signalling [16].

In summary our data suggest that RKIP expression in the cell line UISO is not critical for inactivity of the MAPK pathway, proliferation properties and sensitivity of these MCC cells towards apoptosis inducing agents.
